# Arf6 recruits the Rac GEF Kalirin to the plasma membrane facilitating Rac activation

**DOI:** 10.1186/1471-2121-8-29

**Published:** 2007-07-18

**Authors:** Tae Hyeon Koo, Betty A Eipper, Julie G Donaldson

**Affiliations:** 1 Laboratory of Cell Biology, NHLBI, NIH, Building 50, Rm 2503, Bethesda, MD 20892, USA; 2 Department of Neuroscience, University of Connecticut Health Center, Farmington, CT 06030, USA

## Abstract

**Background:**

Many studies implicate Arf6 activity in Rac-mediated membrane ruffling and cytoskeletal reorganization. Although Arf6 facilitates the trafficking of Rac1 to the plasma membrane and in many cases Arf6 activation leads to the activation of Rac1, the details of how Arf6 influences Rac function remain to be elucidated.

**Results:**

We demonstrate in binding assays and by co-immunoprecipitation that GDP-bound Arf6 binds to Kalirin5, a Rho family guanine nucleotide exchange factor, through interaction with the spectrin repeat region. In cells, expression of wild type Arf6 recruits spectrin repeat 5 and Kalirin to the plasma membrane and leads to enhanced Kalirin5-induced ruffling. By contrast, expression of an Arf6 mutant that cannot become activated, Arf6 T27N, still recruits spectrin repeat 5 and Kalirin to membranes but inhibits Kalirin5-induced ruffling in HeLa cells. Kalirin5-induced Rac1 activation is increased by the expression of wild type Arf6 and decreased by Arf6T27N. Furthermore, expression of a catalytically-inactive mutant of Kalirin5 inhibits cytoskeletal changes observed in cells expressing EFA6, an Arf6 guanine nucleotide exchange factor that leads to activation of Rac.

**Conclusion:**

We show here with over-expressed proteins that the GDP-bound form of Arf6 can bind to the spectrin repeat regions in Kalirin Rho family GEFs thereby recruiting Kalirin to membranes. Although Kalirin is recruited onto membranes by Arf6-GDP, subsequent Rac activation and membrane ruffling requires Arf6 activation. From these results, we suggest that Arf6 can regulate through its GTPase cycle the activation of Rac.

## Background

Arfs are small, ubiquitous, Ras-related GTPases that play an essential role in membrane traffic and structure. Among the 6 mammalian Arf proteins, Arf6 has been implicated in the regulation of endocytic membrane traffic and cortical actin structure at the plasma membrane (PM) [1,2]. The small GTPases of the Rho subfamily are also known to be important regulators of the actin cytoskeleton. To date, at least 25 Rho family proteins have been identified and of these, RhoA, Rac1 and Cdc42 have been the most widely studied for their effects on actin organization. In particular, Rac1 controls the formation of lamellipodia and membrane ruffles, the sites of protrusive actin polymerization and early adhesion formation initiated at the leading edge of migrating cells [3,4].

Recent studies have uncovered crosstalk between these 2 families, in which both Arf6 and Rac proteins have emerged as key regulators in actin remodeling and membrane trafficking events [5-8]. We showed that Rac1 colocalizes with Arf6 at the plasma membrane and recycling endosomes, and that Rac1-stimulated ruffling requires Arf6 activity [9]. Stimulation of the hepatocyte growth factor receptor in MDCK cells [10] or the angiotensin II receptor in Hek cells [11]leads to the sequential activation of Arf6 and then Rac. On the other hand, during platelet activation Arf6-GTP levels decrease, followed by activation of Rho GTPases [12]. Santy and Casanova showed that activation of Arf6 by the guanine nucleotide exchange factor (GEF) ARNO leads to the subsequent activation of Rac [7]. Recently, these investigators found that expression of a dominant negative form of Dock180/ELMO, a Rac GEF, inhibited ARNO activation of Rac suggesting that this Rac GEF may couple ARNO activities to Rac activation [13].

Like other GTPases, both Arf and Rho proteins are regulated by cycling between active, GTP-bound and inactive, GDP-bound conformations. This cycle is regulated by GEFs that catalyze exchange of GTP for GDP and GTPase-activating proteins (GAPs) that catalyze GTP hydrolysis. These regulators control the activation of these GTPases spatially and temporally. Rho family GEFs have a Dbl homology (DH) domain, a conserved amino acid sequence that is the catalytic domain. Many Dbl family GEFs also contain other protein-protein interaction domains that allow GTPases to be activated in specific signal transduction pathways and coordinate more elaborate responses to specific demands at localized cellular sites [14,15].

In the present study, we identified Kalirin, a Rho family GEF, as a specific Arf6 binding protein. Kalirin, a novel member of the Dbl family, is a multi-domain protein with many isoforms in rat adult brain tissues as determined by Northern blot analysis [16,17]. Kalirin5 is the shortest protein of the Kalirin isoforms, containing 5 spectrin-like repeats (SR), a Dbl homology (DH) domain, a pleckstrin homology (PH) domain, and a PDZ binding motif. Kalirin is highly homologous to Trio, which is more widely expressed [18]. Here we provide evidence that Arf6-GDP can bind to Kalirin through interaction with these spectrin repeats, thereby recruiting Kalirin to the membrane leading to Rac activation.

## Results

### Arf6-GDP binds to Kalirin via the spectrin repeats

**Figure 1:**
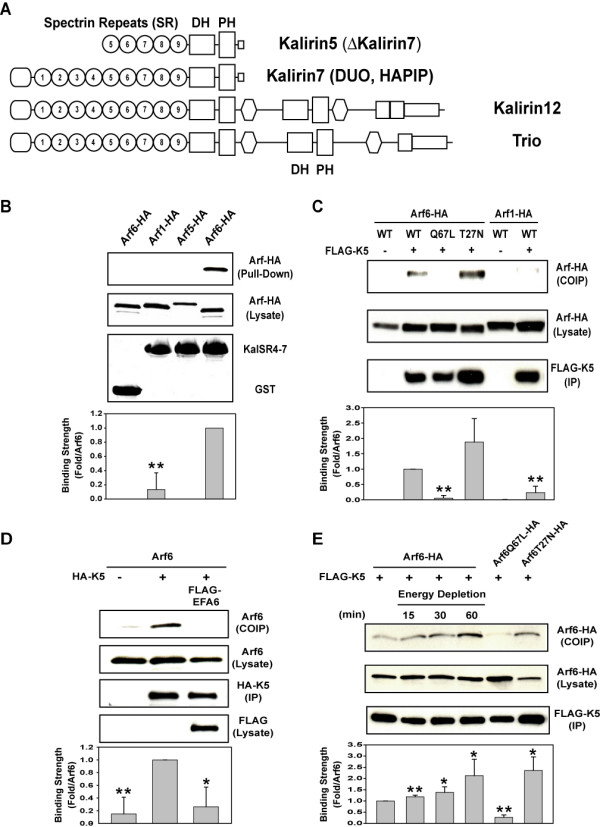
**Kalirin5 interacts with Arf6-GDP**. A. Domain structure of Kalirin isoforms and Trio showing SR, common first and second DH-PH motifs. (Also depicted, Sec14 domain, oval and SH2 domain, hexagon). B. Arf6, but not Arf1 or Arf5, binds to SR region of Kalirin. COS7 cells were transfected with C-terminally HA-tagged Arf isoforms, and cell extracts were incubated with GST-KalSR4-7 or with GST. Arfs were detected by immunoblotting with anti-HA antibody. The GST fusion proteins were visualized by Coomassie staining. C. Arf6-GDP co-immunoprecipitates with Kalirin5. FLAG-tagged Kalirin5 was co-expressed with HA-tagged Arf6, Arf6Q67L, Arf6T27N or with Arf1 in COS7 cells. Lysates were immunoprecipitated with FLAG antibody and then immunobloted with HA antibody to detect Arf proteins. D. Expression of EFA6 decreases the interaction between Arf6 and Kalirin5. FLAG-tagged EFA6 was co-expressed with Arf6 and HA-tagged Kalirin5 in COS7 cells and the cell lysates were immunoprecipitated with HA antibody and then immunobloted as indicated. E. Energy depletion increases the interaction between Arf6 and Kalirin5. COS7 cells co-transfected with HA-tagged Arf6 WT and FLAG-tagged Kalirin5 were incubated with 50 mM 2-deoxyglucose and 0.02 % sodium azide to deplete cellular ATP and GTP levels for 0, 15, 30, or 60 minutes prior to immunoprecipitation. For each panel, the graph summarizes data for 3 independent experiments (mean ± s.d.). Statistical comparison using one-way ANOVA. *p < 0.05 and **P < 0.01.

To identify proteins that interact with Arf6, we used the yeast two-hybrid system to screen a human fetal brain library. One clone was identified that contained the spectrin repeat (SR) domain 5 of Kalirin, a novel member of the Dbl family of Rho GEFs. There are multiple splice forms of Kalirin family GEFs, the longest of which is Kalirin12, a dual Rho GEF homologous to Trio [17,18]. In both Kalirin and Trio, the first DH-PH motif activates Rac and RhoG while the second activates Rho (Fig. 1A) [19,20]. Both Kalirin5 (also called ΔKalirin7) and Kalirin7 (also called Duo) lack the second DH-PH motif, terminating with a PDZ motif; Kalirin5 lacks the Sec14 and first four spectrin repeats of Kalirin7 [17]. We examined whether we could detect a specific interaction of Kalirin5 and its SR with Arf6 by a GST pull-down assay. COS7 cells were transfected with HA-tagged Arf1, Arf5 or Arf6 and the lysates were incubated with spectrin-like repeats 4–7 of rat Kalirin7 (KalSR4-7) fused to GST [21]. Arf6 bound to GST-KalSR4-7 but not to GST alone (Fig. 1B). Moreover, neither Arf1 nor Arf5 bound to GST-KalSR4-7.

We then examined the interaction between Kalirin5 and Arf6 in cells expressing FLAG-tagged Kalirin and various HA-tagged Arf proteins by immunoprecipitation of FLAG-Kalirin. Wild type Arf6 and the GTP-binding defective mutant of Arf6 (Arf6T27N) co-immunoprecipitated with Kalirin5 but a constitutively active mutant of Arf6 (Arf6Q67L) did not (Fig. 1C). Neither Arf1 nor Arf5 co-precipitated with Kalirin, consistent with the results in the GST pull-down assays. Similar results were obtained when Arf proteins were immunoprecipitated with antibody to HA and the immunoprecipitates probed for Kalirin5 (data not shown). Since cells expressing Arf6 will have both GDP and GTP-bound forms, whereas cells expressing Arf6T27N will have only the GDP-bound form of Arf6, these results suggest that Kalirin5 binds to the GDP-bound, and not the GTP-bound, form of Arf6. Although we could see this interaction following over-expression of Arf6 and Kalirin, we were not able to detect it with endogenous proteins; this may be due to low levels of expression, difficulty extracting the proteins in a native state and lack of sensitive immunological reagents.

To confirm this Arf6-GDP-specific interaction, we co-expressed Arf6 and Kalirin5 with EFA6, an Arf6 GEF that will lead to an increase in Arf6-GTP [22,23] and hence a decrease in Arf6-GDP in cells. Co-expression of EFA6 decreased the amount of Arf6 recovered in the Kalirin5 immunoprecipitate (Fig. 1D). Secondly, lowering ATP and GTP levels in cells by treatment with sodium deoxyglucose and sodium azide, used previously by others [24,25], led to a time-dependent increase in Arf6 and Kalirin5 binding (Fig. 1E). Enhanced Arf6 binding to SR5 in the GST-spectrin pull down assays was also observed when cells were depleted of energy as above (data not shown). Similar co-immunoprecipitation results were obtained from transfected HeLa cells (data not shown). We also attempted co-immunoprecipitation of larger Kalirin-like isoforms (Kalirin7 and 12) but were unsuccessful due to a very low efficiency of transfection of these large SR-containing GEFs. From these results, we conclude that Kalirin5 can bind specifically to Arf6-GDP.

**Figure 2:**
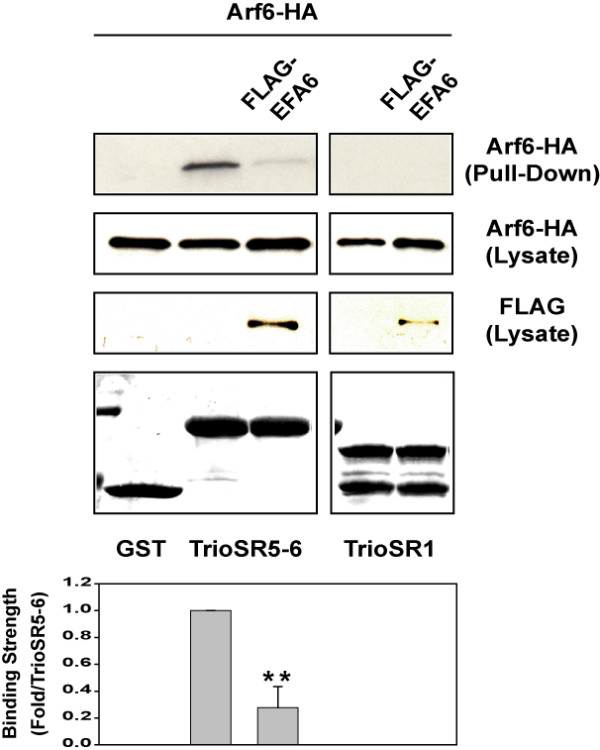
**SR5 from Trio can interact with Arf6**. COS7 cells were transfected with HA-tagged Arf6 with or without FLAG-tagged EFA6. Cell extracts were incubated with GST-tagged TrioSR5-6 or TrioSR1. Complexes were immobilized on glutathione-Sepharose, extensively washed, and separated by SDS-PAGE together with aliquots of total cell lysates. Detection of bound protein was performed by immunoblotting with anti-HA antibody. The bound GST fusion proteins were visualized by Coomassie staining. Graph summarizes data for 3 independent experiments (mean ± s.d.); **p < 0.01 versus Arf6 and Trio SR5-6 (one way ANOVA).

Trio has a similar domain structure to that of Kalirin (Fig. 1A) [17,18]. In particular, the amino acid identity between Trio and Kalirin in their spectrin repeat regions is high; the SR5 regions are 69% identical. Since Trio is expressed in HeLa cells [18], we examined whether Arf6 could interact with Trio via its SR region. Arf6 interacted with GST-Trio SR5-6 and co-expression of EFA6 diminished this interaction (Fig. 2). Additionally, we did not see any Arf6 binding to Trio SR1 (Fig. 2). Thus, SR5 may represent a specific domain for interaction with Arf6-GDP and Trio function may also be regulated by Arf6.

### Arf6 regulates the cellular localization of Kalirin5

**Figure 3:**
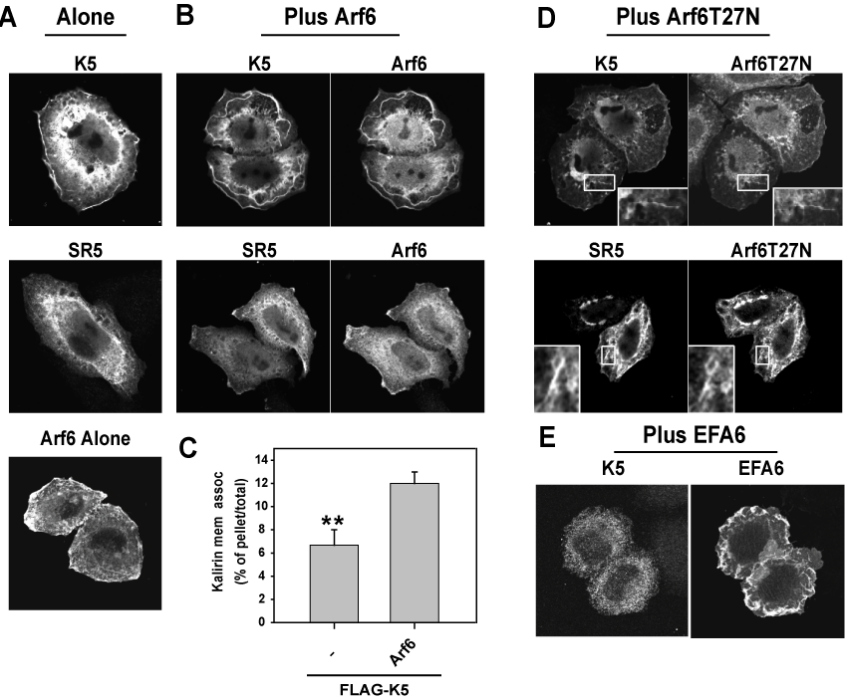
**Arf6 recruits Kalirin5 to the PM and endosomes and enhances ruffling**. A, B, D, E. Expression of Kalirin5 (K5), SR5 or Arf6 alone (A) or co-expressed with wild type Arf6 (B), Arf6T27N (D) or EFA6 (E). HeLa cells were transfected with FLAG-tagged Kalirin5 or SR5 alone, or with wild type Arf6, Arf6T27N or EFA6. After 18 h, cells were washed, fixed, and processed for indirect immunofluorescence to detect the localization of the overexpressed proteins. C. Increased association of Kalirin with membrane fraction in Cos7 cells expressing Arf6. Rapid fractionation of membrane and cytosol was performed on cells expressing FLAG-Kalirin5 alone or co-expressed with Arf6. The percentage of total Kalirin that was recovered in the membrane pellet is shown for three independent experiments (mean ± s.d.). **p < 0.01 versus Arf6 and Kalirin5 co-transfectants (one way ANOVA).

Having shown that Kalirin5 binds specifically to Arf6-GDP *in vitro*, we next examined the intracellular distribution of Kalirin5 and various spectrin repeat constructs in transfected HeLa cells. We used HeLa cells to assess the distribution of these proteins and their effects on cell morphology because we have a good understanding of Arf6 and Rac function in these cells [9,23]. Expression of FLAG-tagged Kalirin5 alone induced modest ruffling (Fig. 3A). Kalirin5 was observed at the PM and also diffusely throughout the cytoplasm. Kalirin5-induced ruffling was dependent upon Rac1 since coexpression with Rac1T17N inhibited ruffling (data not shown). Expression of SR5 or Arf6 alone did not cause PM ruffling (Fig. 3A). Co-expression of Arf6 with Kalirin5 greatly enhanced the formation of membrane ruffles (Fig. 3B), consistent with previous observations that Rac1-mediated ruffling is enhanced by Arf6 expression in HeLa cells [9]. We also observed that Arf6 co-expression increased the association of Kalirin with the PM, where the two proteins colocalized and decreased the cytoplasmic pool of Kalirin (Fig. 3B). When expressed alone in HeLa cells, SR5 was primarily cytoplasmic (Fig. 3A), but co-expression with Arf6 recruited SR5 to the PM where it colocalized with Arf6 (Fig. 3B).

This increased membrane association of Kalirin in the presence of Arf6 expression was also shown by subcellular fractionation; COS7 cells, which show a higher co-transfection rate than do HeLa cells, were used for these studies. While only about 7% of Kalirin5 was associated with the membrane fraction when expressed alone, this amount nearly doubled when Arf6 was co-expressed (Fig. 3C).

When Arf6T27N was expressed with Kalirin5, ruffling was inhibited and the amount of Kalirin5 at the PM was reduced. Kalirin colocalized with Arf6T27N on the tubular endosomal membranes (Fig. 3D inset), previously shown to carry recycling membrane cargo such as MHCI back to the PM [26,27]. The PM localization of SR5 was also diminished in cells expressing Arf6T27N (Fig. 3D); SR5 was, instead, colocalized with Arf6T27N on tubular endosomal membranes (Fig. 3D insets). In contrast to its recruitment to membranes in cells expressing Arf6T27N, Kalirin5 was less evident at the PM in cells coexpressing EFA6, which causes an increase in Arf6-GTP (Fig. 3E). The recruitment of Kalirin to membranes by expression of the GDP-bound mutant of Arf6 (T27N), and not by expression of EFA6, supports the biochemical interaction data between Arf6-GDP and Kalirin5 (Fig. 1C–E). Similar results were observed in COS7 cells (data not shown).

### Arf6 affects Rac1 activity through Kalirin5

**Figure 4:**
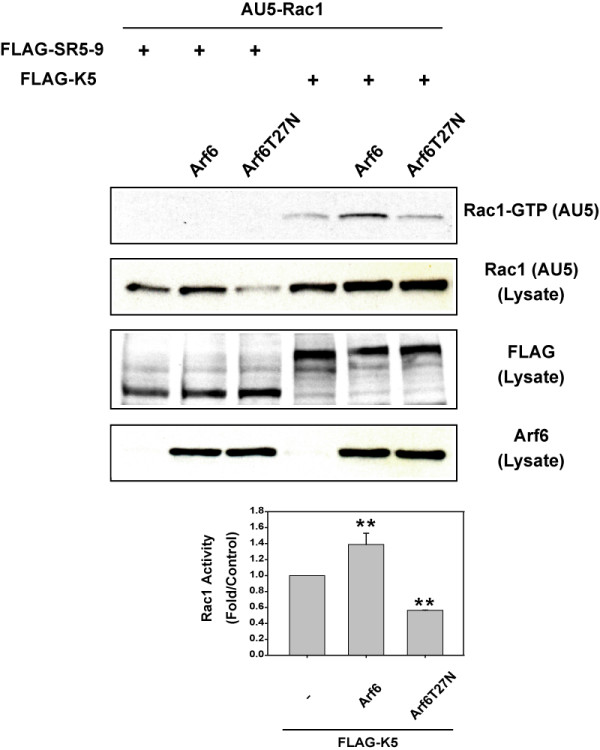
**Arf6 increases Kalirin5-induced Rac1 activation**. COS7 cells were co-transfected with AU5-tagged Rac1 and with FLAG-tagged SR5-9 or FLAG-tagged Kalirin5 and with Arf6 or Arf6T27N. For each sample, total cell lysates were incubated with immobilized GST-Pak-CRIB and bound AU5-Rac (AU5-Rac-GTP) was detected by immunoblotting. Total cell lysates were also analyzed by immunoblotting. Graph depicts amount of AU5-Rac1-GTP relative to that observed in Kalirin 5 expressing cells. Mean ± s.d. of three independent experiments; **p < 0.01 versus Kalirin5 transfectants (one way ANOVA).

Since Kalirin5-induced ruffling was dependent upon Rac1 in HeLa cells, we examined whether we could detect Rac activation in a pull-down assay using the CRIB domain of Pak1 fused to GST [28]. Total cell lysates from cells expressing AU5-tagged Rac1 were incubated with GST-Pak-CRIB and bound Rac1 (Rac1-GTP) was detected by immunoblotting. No Rac1-GTP was detected in cells expressing Rac1 plus SR5-9 and this did not change with co-expression of Arf6 (Fig. 4). Co-expression of Kalirin5, however, led to detectable Rac1-GTP that was increased further upon co-expression of Arf6. In contrast, co-expression of Arf6T27N diminished the ability of Kalirin5 to activate Rac1 (Fig. 4). Even though the extent of Rac1 activation was modest, likely due to low efficiency of co-expression, these results suggest that wild type Arf6 enhances the ability of Kalirin5 to activate Rac1 whereas an Arf6 mutant that cannot be activated, Arf6T27N, diminishes Kalirin5 activation of Rac1.

**Figure 5:**
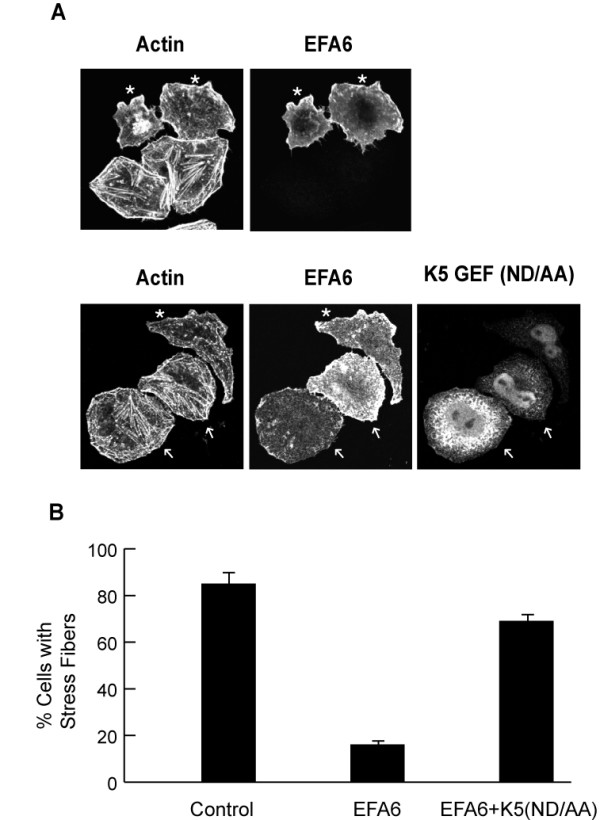
**Dominant negative Kalirin5 (ND/AA) inhibits EFA6 changes in actin organization**. A. HeLa cells were transfected with FLAG-tagged EFA6 alone or with HA-tagged Kalirin5 (ND/AA). After 24 h, cells were fixed and labeled with antibodies to detect EFA6, Kalirin and with rhodamine phalloidin to detect actin. Asterisks indicate cells expressing EFA6 only. Arrows indicate cells co-expressing EFA6 and Kalirin5(ND/AA). B. Quantification of cells with radial stress fibers in control, untransfected cells and in cells transfected with EFA6 alone and EFA6 and Kalirin5 (ND/AA). Means ± s.d. of three independent experiments.

Finally, to support a model whereby Arf6 affects Rac activation through recruitment of an endogenous SR-containing GEF, possibly Trio, in HeLa cells, we examined whether a dominant inhibitory mutant in the DH domain of Kalirin5 (N813A/D814A) [29,30] could block the alterations in the cytoskeleton induced by EFA6, an Arf6 GEF. Expression of EFA6 leads to protrusive cells with a marked increase in cortical actin and marked decrease in stress fibers (Fig. 5A); this response requires activation of Arf6 and subsequent activation of Rac [22,23]. Co-expression of mutant Kalirin5 (ND/AA) blocked these changes in cell shape and actin polymerization normally observed in cells expressing EFA6 (Fig. 5A cells marked with arrows) whereas co-expression of wild type Kalirin5 with EFA6 did not inhibit EFA6 PM ruffling (see Fig. 3E). Cells with stress fibers, scored by exhibition of radial fibers crossing the cell center, were observed in only 20% of EFA6 expressing cells but this was increased to 70% in cells co-expressing the Kalirin5 mutant (Fig. 5B). This is similar to the 85% frequency of cells exhibiting radial stress fibers in untransfected cells (Fig. 5B). We could not measure the expected decrease in EFA6-induced Rac activation biochemically in the presence of GEF-dead Kalirin5 due to the low frequency of cells expressing all three proteins.

## Discussion

Many studies have demonstrated a role for Arf6 activity in Rac-mediated membrane ruffling and cytoskeletal reorganization at the PM. Arf6 may facilitate the trafficking of Rac1 to the PM [5,9,31] and Arf6 activation may lead to the activation of Rac1 [7]. However, the details of how Arf6 influences Rac functions remain to be elucidated. A recent study by Santy et al has shown that the ARNO activation of Arf6 that leads to the activation of Rac in MDCK cells may be mediated by the DOCK180/Elmo complex however, no direct interaction between ARNO or Arf6 and DOCK180/Elmo was described [13]. In the present study, we describe a possible mechanism for how Arf6 could regulate Rac1 activity by showing the interaction between Arf6-GDP and Kalirin5, a Rac/RhoG GEF.

Kalirin5 is the smallest isoform in the Kalirin family. The amino terminal end of the protein begins at spectrin repeat 5 (SR5) and includes the Dbl homology (DH) and pleckstrin homology (PH) domains and a PDZ-binding motif in the C-terminal region (Fig. 1). Most of the alternatively spliced isoforms of both Kalirin and Trio include these spectrin-like repeats [18,32]. Interestingly, these spectrin repeats are also sites of interaction of Kalirin with PAM, a secretory granule enzyme required for the α-amidation of peptides [16], with i-NOS [33], and with Huntingtin-associated protein 1 (HAP1) [34]. Since the larger Kalirin isoforms all contain SR5, they could interact with Arf6-GDP in a manner similar to Kalirin5. We do not know whether this binding is direct or involves other proteins.

What then could be the function of Arf6-GDP binding to Kalirin? We propose that the Kalirin interaction with Arf6-GDP is transient but brings Kalirin to membranes. The subsequent activation of Arf6 then allows Kalirin to activate Rac. Consistent with this, co-expression of wild type Arf6, which can cycle between GDP-and GTP-bound forms, increases the PM localization of Kalirin5, the steady state level of Rac1-GTP, and the ability of Kalirin5 to induce membrane ruffling. Arf6T27N, however, being trapped in the GDP-bound state, recruits Kalirin5 to endosomal membranes but fails to allow activation of Rac through Kalirin. On the other hand, EFA6 promotes activation of Arf6 but still allows inactivation, generating Arf6-GDP. When EFA6 is expressed, Kalirin does not stably associate with the PM but it may transiently be brought to the PM by Arf6-GDP to activate Rac. The ability of GEF dead Kalirin5 to block EFA6-induced cytoskeletal changes (Fig. 5) is supportive of this model and suggests that a spectrin repeat-containing GEF might be responsible for Rac activation in EFA6 expressing cells.

Kalirin brought to the membrane through its interaction with Arf6-GDP could be positioned for optimal access to Rac1. Additionally, the interaction of Arf6 with SR5 of Kalirin could allosterically activate its GEF activity. In either case, the interaction of Arf6 with Kalirin provides a way to couple the Arf6 activation/inactivation cycle to that of a Rho GTPase. The sequential recruitment of Kalirin by Arf6-GDP, followed by activation of Arf6 and then activation of Rac is consistent with earlier observations of the requirement of Arf6 activation for Rac ruffling, the synergy between Arf6 and Rac [9] and the fact that activation of Arf6 leads to activation of Rac [7]. Furthermore, the requirement that Arf6 cycle between GDP-bound and GTP-bound forms to carry out its cellular functions is consistent with the known biology of Arf proteins [1].

Intriguingly, two examples of proteins binding to Arf6-GDP that subsequently lead to Arf6 activation have been reported. TRE17, a protein involved in Rac activity, binds specifically to the GDP-bound form of Arf6, which leads to increased activation of Arf6 [24] presumably by bringing Arf6 in proximity to an Arf6 GEF. The second example is β-arrestin, a regulator of agonist-stimulated, β2-adrenergic receptor endocytosis, which binds to both Arf6-GDP and ARNO, an Arf6 GEF, leading to activation of Arf6 [35]. Like β-arrestin, Kalirin5 can make a complex with Arf6-GDP and perhaps lead to the recruitment of EFA6 or ARNO to promote Arf6 activation. Indeed, we have been able to capture Arf6T27N and EFA6 together bound to immobilized SR5 (unpublished observations) suggesting that Kalirin may provide a scaffold for sequential and coordinated activities.

Our studies demonstrate that Arf6 can regulate Rac1 activity through interaction with Kalirin. This novel signaling pathway may be involved in neurite outgrowth since each of these proteins are abundant in brain and have been implicated in this process. Arf6 activity is required for Rac1-mediated neurite outgrowth [36] and the GEF1 domains of Kalirin and Trio also induce neurite outgrowth through RhoG activation [37,38]. Exploring this functional relationship between Arf6 and Kalirin will give us more information about the mechanism for Rho GEF regulation and the crosstalk between these two small GTPases, Arf6 and Rac.

## Conclusion

The GDP-bound form of Arf6 can recruit spectrin repeat-containing Rho GEFs, such as Kalirin, to membranes. The requirement that Arf6 be able to cycle between GDP and GTP-bound forms for Rac activation reveals how the Arf6 GTP cycle may coordinate Rac function.

## Methods

### Cells, plasmids and transfection

HeLa and COS7 cells were maintained in Dulbecco's modified Eagle's medium (DMEM) supplemented with 10% fetal bovine serum (FBS) at 37°C with 5% CO_2_. Rabbit polyclonal antibody to Arf6 was described elsewhere [39]. Mouse monoclonal antibodies to AU5 and HA was from Covance (Berkeley, CA) and to Rac was from Upstate (Lake Placid, NY). All secondary antibodies conjugated to Alexa 594 and 488 were from Molecular Probes (Eugene, OR). To clone human Kalirin5, polymerase chain reaction (PCR) was performed with human fetal brain cDNA library (Clontech, Palo, CA) as a template. The GEF domain point mutant of human Kalirin5 (N813A, D814A) was generated by site-directed mutagenesis (Stratagene, La Jolla, CA). All constructs were verified by DNA sequencing. Glutathione S-transferase (GST)-rat Kalirin7 spectrin repeats 4–7 [21], human Trio spectrin repeat 1 and human Trio spectrin repeats 5–6 [32] were as described. GST-Pak1-CRIB and AU5-tagged Rac1 WT were from Dr. J. Silvio Gutkind (NIH, Bethesda, MD). Arf6 and mutants are in pXS plasmid [9]; FLAG-EFA6 is in pFLAG-CMV [23]. For transfection, cells were plated and transfected the next day using FuGene 6 (Roche Diagnostics, Indianapolis, IN). Experiments were performed 18–24 h after transfection.

### GST pull-down experiments

COS7 cells were transfected with plasmids encoding HA-tagged Arf1, Arf5 or Arf6. Twenty four hours later, cells were washed three times with ice-cold PBS and extracted in 0.5 ml of ice-cold lysis buffer [25 mM HEPES (pH 7.5), 150 mM NaCl, 0.25% NP-40, 0.1% sodium deoxycholate, 10% glycerol, 1 mM MgCl_2_, 1 mM Na_3_VO_4_, 20 mM NaF, 1 mM 4-(2-aminoethyl)-benzenesulfonyl fluoride, 2 μg/ml leupeptin, 2 μg/ml aprotinin, 2 μg/ml pepstatin, 1 mM DTT]. Cell extracts were incubated with GST or GST-spectrin repeats of Kalirin or Trio for 60 min at room temperature, washed and 75% of bound proteins and 2% of total cell lysate were separated by SDS-PAGE and analyzed by Western blot analysis using Odyssey infrared imaging system (Li-Cor, Lincoln, NE).

### Immunoprecipitation

Cell transfection and lysis conditions were similar to those used in the GST pull-down experiments (see above). Lysates from Cos7 cells were mixed with 10 μg of anti-FLAG M2 monoclonal antibody (Sigma) and then incubated overnight at 4°C with gentle rotation with 15 μl of Protein G Sepharose beads (Amersham, Piscataway, NJ). The beads were washed three times in lysis buffer and then boiled in 1× SDS sample buffer and 75% of bound material and 2% of the total cell lysate were separated by SDS-PAGE and analyzed by Western blot analysis using Odyssey infrared imaging system.

### Immunofluorescence

Cells were plated on glass coverslips and transfected the next day. 18–24 h after transfection, the cells were fixed in 2% formaldehyde for 10 min and processed for immunofluorescence as described [26]. All images were obtained using a 510 LSM confocal microscope (Carl Zeiss, Thornwood, NJ) with 63× Plan Apo objective. Acquisition of figures was accomplished in Adobe Photoshop 5.5.

### Fractionation

For fractionation, cells were processed as described [39]. Briefly, cells were scraped into ice-cold PBS and pelleted at 300 × ***g ***for 5 minutes at 4°C and then resuspended in ice-cold 250 mM sucrose/10 mM Tris-HCl, pH 7.4. The cells were lysed by passing the cell suspension 5 times through a 25-gauge needle attached to a 1 cc syringe. After low speed centrifugation to remove unbroken cells and nuclei, the post-nuclear supernatant was subjected to centrifugation at 100,000 × ***g ***for 30 minutes at 4°C in a TLA100 rotor (Beckman, Fullerton, CA). Sedimented material was resuspended in SDS sample buffer to the same volume as the supernatant. Proportional amounts of both samples were separated by SDS-PAGE and analyzed by Western blot.

### Rac1 activation assay

Activation of Rac1 was determined by measuring the amount of AU5-Rac1 bound to the CRIB domain of Pak coupled to GST (GST-Pak1-CRIB)[28]. COS7 cells were transfected with plasmids encoding AU5-tagged Rac1, with or without ARF6 (wild type or T27N), FLAG-tagged SR5-9, or FLAG-tagged Kalirin5. Twenty four hours later, cells were washed twice with serum-free DMEM and then serum starved for 4 h. The cells were then lysed at 4°C in buffer containing 25 mM HEPES, pH 7.5, 250 mM NaCl, 1 mM EDTA, 10 mM MgCl_2_, 1 % Igepal CA-630, 2 % glycerol, 1 mM 4-(2-aminoethyl)-benzenesulfonyl fluoride, 2 μg/ml leupeptin, 2 μg/ml aprotinin, 2 μg/ml pepstatin and 1 mM DTT. Lysates were then incubated with GST-Pak1-CRIB beads for 1 h at 4°C. The beads were washed three times in lysis buffer and then boiled in 1× SDS sample buffer and separated by SDS-PAGE and analyzed by Western blot using antibody to AU5.

### Statistical methods

Statistical comparison was made using one-way ANOVA. Values are expressed as the mean ± standard deviation of three independent experiments performed in triplicate. P values < 0.05 were considered significant and < 0.01 were considered very significant.

## Abbreviations

DH, Dbl homology; GEF, guanine nucleotide exchange factor; PH, pleckstrin homology; PM, plasma membrane; SR, spectrin repeats.

## Authors' contributions

THK helped conceive of the study, carried out the experimental work, helped analyze the data and wrote the first draft of the manuscript. BAE helped conceive of the study, performed some immunoprecipitations, helped analyze the data and participated in revising the manuscript. JGD helped conceive of the study, participated in the design and coordination of the experiments, analyzed the data and revised the manuscript.
